# Statistics and Their Critics: King Edward's Hospital Fund for London Statistics—Fourth Notice


**Published:** 1915-02-06

**Authors:** 


### STATISTICS AND THEIR CRITICS.

### King Edward's Hospital Fund for London Statistics.—Fourth Notice.*

A critical and able correspondent of The Hospital, in a letter dated October 14, 1914, appealed to us to strive against becoming the slaves of statistics. He urges that mere figures without an intimate knowledge of facts are of little value, and that in the preparation of accounts under the Uniform System too much latitude is given to the accountant, and consequently there is a temptation to present a pleasing picture while still keeping within the ample limits of statistical accuracy afforded by the system. If the figures as presented are, he argues, a sufficient guide to the public, how can the £90 per occupied bed hospital evade the odium of extravagance and the £60 successfully survive a charge of parsimony, for both, on economical lines, cannot claim perfection in methods of administration?

The writer of the letter, regarding the matter from a point of view very different from that of the editor of the Statistical Report, naturally makes the most of every point in support of his arguments. His idea, that King Edward's Fund should slacken the disciplinary control they yield through the power of the purse, and employ their influence instead in popularising hospitals, is reactionary in that it is entirely opposed to all modern methods of public assistance, and reverts to the systems of the bad old days of last century. The fund, or the person who gives without inquiry, frequently does harm where good is intended. The hospital administrator rightly does not despise any reputable form of support, but the friends he chiefly strives to make are the constant level-headed supporters who will stand beside him at all times, not those who are swayed by the sentiment of the moment. The recognition and approval of the King's Fund, gained, as the public knows, after a careful scrutiny of administration, is exceedingly valuable in obtaining the support of the careful giver, who, in the long run, is the generous giver and more valuable than the subscriber who requires constant stimulation of his sentimental nature.

Some of the points raised by the correspondent of October 14 have obviously not escaped the advisers of the King's Fund, who, with commendable patience, have directed their attention to those who have evaded the directions contained in previous editions of the Red Book of instructions for preparing accounts through what would appear to be purposeful stupidity, and who find their evasions made impossible by elaborate instructions in subsequent issues. For instance, the accountant who calculated one attendance of an out-patient as four because the patient took away medicine for a month can repeat his ingenious operation no longer. The hospital (instanced by our correspondent) that always deducted the cost of an appeal from the result, even if this course were ever justified (which we doubt), can do so no more in the face of paragraph 10 on page 6 of the third edition of the Red Book. Where we must agree with the critic is in respect of his remarks on undisclosed information as to special influences on cost.

As readers of former articles on this subject in The Hospital know, the presentation of all essential information in connection with each institution has been constantly advised, and the warning given by the issuers of the Statistical Report, that too hasty conclusions must not be formed on the basis of the figures alone, has been repeated in these columns. It is common knowledge that committees of management vary widely in their ideas of what is good enough for the patient and what remuneration and living conditions are adequate for the staff. These things have, we think, a way of levelling themselves in time. Insufficient feeding of patients is uneconomical, as it must produce its mark on the medical statistics and an unenviable reputation for the institution. Low conditions for the staff will inevitably result in a low standard of service. If the £60 per bed hospital possesses these faults, "economical" is an incorrect adjective to use in reference to an administration with which true economy is in no way associated.

The Statistical Report, if we have read it intelligently, militates quite as much against insufficient as excessive expenditure, and the whole history of King Edward's Hospital Fund is full of evidence that efficiency is not only expected, but encouraged by liberal grants to schemes for improving hospitals in every respect and bringing about better conditions for the benefit of patient and staff, which in many cases must add to annual cost.

### A Practical Example.

We know that many people give carelessly to charities. A philanthropist may be swayed by whether he likes a £60 or a £90 a bed hospital, but it is by no means certain that, in the absence of obviously undisclosed information, he will favour the cheaper of the two. There is a careful giver who is always welcome to a conscientious administration; he is the one who asks questions, who visits the institution, and often becomes a life-long and generous supporter. If his interest has been aroused or stimulated by the King's Fund returns, that is but one example of the good brought about by the labours of the compilers of the figures.

But how much may we learn without the undisclosed factors, or rather without the general knowledge of these factors, which is not already in the possession of an intelligent student of hospital affairs? Let us take three hospitals (the only common similarity of which is in respect of size) and endeavour to criticise the figures from a detached point of view aided only by a general knowledge of hospital administration and the information afforded by the Statistical Report. We will set out the main particulars on the table overleaf.

* Previous articles appeared on October 10 and December 12, 1914, and January 23, 1915.

Cost per Bed of Three Similar-sized Hospitals.

| Nature of Hospital | Number of Occupied Beds | Number of Out-Patient Attendances | Total Ordinary Expenditure | Cost per Occupied Bed Under Headings: Salaries and Wages Maintenance | Provisions | Surgery and Dispensary. | Domestic | Management Administration | Total | Cost per Occupied Bed of Whole Group of Similar Hospitals | Cost per Out-Patient Attendance | Cost per Out-Patient Attendance of Whole Group of Similar Hospitals |
|---|---|---|---|---|---|---|---|---|---|---|---|---|
| | | | £ | £ s. d. | £ s. d. | £ s. d. | £ s. d. | £ s. d. | £ s. d. | £ s. d. | d. | d. |
| (A) For Diseases of the Chest ......... | 163 | 31,391 | 13,994 | 18 13 8 | 24 18 4 | 2 4 3 | 13 17 2 | 1 7 5 | 61 0 10 | 66 17 4 | 12 | 15.50 |
| (B) For Diseases of the Nervous System ............ | 153 | 43,988 | 16,879 | 26 8 3 | 25 2 9 | 4 0 6 | 18 12 2 | 1 1 4 | 75 5 0 | 68 14 6 | 9.74 | 8.44 |
| C) A General Hospital Without a Medical School... | 176 | 80,437 | 20,536 | 25 2 4 | 24 9 9 | 10 4 11 | 19 8 5 | 1 4 8 | 80 10 1 | 84 11 2* | 7.68 | 8.22 |

* King's Fund figures for seventeen larger general hospitals. The hospitals without medical schools taken alone work out at £70 13*s.* 1*d.* per occupied bed.

It will be noticed that in the dissection of the cost per occupied bed we have placed Salaries and Wages—Maintenance first. For the purposes of critical examination of the figures the particulars under this heading are by far the most important, as they have a heavy influence on the headings Provisions and Domestic, and a smaller one on Surgery and Dispensary and Management—Administration. The amount spent on salaries and wages is the only index afforded us to that important factor—the size of the staff. That detailed information on this point should be given in the Statistical Report we have urged more than once. To speak of a hospital having 150 occupied beds means that the daily average of beds occupied by the patients is 150, that there are 150 people to feed, to attend medically, to nurse, to wash for, to tend generally every day. The statement gives no indication of the number of other inmates of the institution who, in addition, require several of these services. These may number fifty, possibly 100; the extent of the establishment is, at present, left entirely to the imagination or knowledge of the reader of the Statistical Report.

We see, for instance, that hospital A is treating patients whose conditions are probably not acute (a hospital for consumption would very properly take the cases it can do most good to—those in the earlier stages of the disease), and possesses no surgical beds. The cost of nursing is therefore much lower than in a nerve hospital with its greater demands in respect of treatment by massage and electricity, and its brain and spine surgery. When compared with hospital C, with 50 per cent. or more of surgical patients and its acute medical cases, the same remark applies. Fewer nurses have to be fed, clothed, and washed for; also the staff of domestic servants and porters may be expected to be smaller, and the expenditure under the headings of Domestic and Provisions correspondingly lighter. But the cost of provisions is almost the same in the average of the three hospitals. As our mission is to criticise and not to justify, we should say that we can understand this in respect of the comparison of the chest hospital, where liberal feeding is part of the treatment, and the general hospital, providing for more people in the aggregate, where, however, a patient with a fracture or some simple medical ailment requires but a plain and sufficient diet. The two conditions might easily balance. In the case of the nerve hospital, taking into consideration its larger staff, the cost seems too low and needs that personal examination which no survey of the figures can supply, because the other hospitals in the nerve group (lower though they are in this particular) make comparison unprofitable by reason of their great divergence in size.

Under Surgery and Dispensary, in view of the large difference between the three hospitals, all our imagination and general knowledge are needed. The consumptive in-patient will require little medicine and next to no dressings, milk and other foods will largely take the place of the cod-liver oil and malt he would be given if he were an out-patient; the requirements of his treatment have their chief effect on the cost of food. But the nervous patients (including the epileptic, for whom has been prescribed bromide or another expensive drug) must be expected to cost more. The patients at the general hospital, if medical, will be expensive under drugs, and if surgical, additionally costly in respect of dressings. But the expenditure of hospital C is remarkably high in comparison to the general average in the group of larger general hospitals, and the reason is not apparent from the Statistical Report or from any consideration arising out of general knowledge of hospital matters.

As our readers will remember, the average cost under Management — Administration excludes official salaries and pensions, but covers postage, printing, stationery, advertisements, auditor's fees, and sundry official expenditures. This being so, the average of hospital A from the particulars supplied appears heavy.

The out-patient expenditure of the three hospitals seems to call for little remark, and in each case compares very well with the corresponding cost of the respective groups to which the institutions belong. One shilling per attendance for the consumption hospital, serving out large quantities of cod-liver oil and extract of malt, is not excessive; considering the expensive dispensary requirements of cases of nervous diseases, 9.74*d.* for hospital B is comparatively low, but the average of the general hospital (C) is so modest that we wonder, taking into consideration the high in-patient cost, if an equitable division has been made.

